# Patient Perspectives and Preferences for Consent in the Digital Health Context: State-of-the-art Literature Review

**DOI:** 10.2196/42507

**Published:** 2023-02-10

**Authors:** Iman Kassam, Daria Ilkina, Jessica Kemp, Heba Roble, Abigail Carter-Langford, Nelson Shen

**Affiliations:** 1 Campbell Family Mental Health Research Institute Centre for Addiction and Mental Health Toronto, ON Canada; 2 Institute of Health Policy, Management and Evaluation University of Toronto Toronto, ON Canada; 3 Canada Health Infoway Toronto, ON Canada

**Keywords:** consent, electronic consent, eConsent, personal health information, patient engagement, digital health, health IT, privacy, eHealth, data sharing, artificial intelligence

## Abstract

**Background:**

The increasing integration of digital health tools into care may result in a greater flow of personal health information (PHI) between patients and providers. Although privacy legislation governs how entities may collect, use, or share PHI, such legislation has not kept pace with digital health innovations, resulting in a lack of guidance on implementing meaningful consent. Understanding patient perspectives when implementing meaningful consent is critical to ensure that it meets their needs. Consent for research in the context of digital health is limited.

**Objective:**

This state-of-the-art review aimed to understand the current state of research as it relates to patient perspectives on digital health consent. Its objectives were to explore what is known about the patient perspective and experience with digital health consent and provide recommendations on designing and implementing digital health consent based on the findings.

**Methods:**

A structured literature search was developed and deployed in 4 electronic databases—MEDLINE, IEEE Xplore, Scopus, and Web of Science—for articles published after January 2010. The initial literature search was conducted in March 2021 and updated in March 2022. Articles were eligible for inclusion if they discussed electronic consent or consent, focused on the patient perspective or preference, and were related to digital health or digital PHI. Data were extracted using an extraction template and analyzed using qualitative content analysis.

**Results:**

In total, 75 articles were included for analysis. Most studies were published within the last 5 years (58/75, 77%) and conducted in a clinical care context (33/75, 44%) and in the United States (48/75, 64%). Most studies aimed to understand participants’ willingness to share PHI (25/75, 33%) and participants’ perceived usability and comprehension of an electronic consent notice (25/75, 33%). More than half (40/75, 53%) of the studies did not describe the type of consent model used. The broad open consent model was the most explored (11/75, 15%). Of the 75 studies, 68 (91%) found that participants were willing to provide consent; however, their consent behaviors and preferences were context-dependent. Common patient consent requirements included clear and digestible information detailing who can access PHI, for what purpose their PHI will be used, and how privacy will be ensured.

**Conclusions:**

There is growing interest in understanding the patient perspective on digital health consent in the context of providing clinical care. There is evidence suggesting that many patients are willing to consent for various purposes, especially when there is greater transparency on how the PHI is used and oversight mechanisms are in place. Providing this transparency is critical for fostering trust in digital health tools and the innovative uses of data to optimize health and system outcomes.

## Introduction

### Background

Digital health refers to the use of IT, services, and processes to support health care delivery [1]. These technologies include but are not limited to electronic health records (EHRs), consumer wearable devices, mobile apps, remote patient monitoring, artificial intelligence (AI), and virtual care [1-3]. Digital health tools can support improved patient engagement and empowerment and enhance care quality and delivery [4,5]. The success of these tools hinges on their ability to share patient data (herein referred to as personal health information [PHI]) to support clinical care [6], enable patient access to health records [7], and progress research and health system analytics (ie, secondary use) [8]. Opportunities for using digital health tools continue to grow as the potential for applications of AI, machine learning, and deep learning expands. Various types of AI are currently being used to support the development of learning health care systems [9]. With the growing volume of data produced through digital health tools, there is considerable interest in consolidating these data silos [10-12]. Numerous health care organizations aim to establish learning health systems to achieve these benefits; however, this work cannot be done without patients’ consent to share their PHI [13]. These systems require large amounts of PHI to develop algorithms that can guide improvements in patient safety, quality of care, and health outcomes [9].

### Digital Health and Patient Privacy

The growing importance of and interest in integrating AI and other digital health tools into care raises questions on how to protect patient privacy. Although the public is supportive of investments in these technologies [14,15], there is also a corresponding concern about the potential for unethical or harmful uses of their PHI [16-18], especially as it relates to use by commercial entities. As health care data breaches and instances of misuse of PHI by commercial entities become more common [19], there is a need to support individuals in better understanding what they are consenting to (ie, becoming informed data citizens). It is necessary to reach a balance between protecting patient privacy and realizing the benefits of digital innovations to support meaningful consent and see success in this space. This balancing act is a product of polarizing perspectives grounded in the different values of various stakeholders in the health care community [20]. Privacy as contextual integrity [21] suggests that the appropriateness of information sharing should be based on the norms of specific social contexts, and trust in a product or service is predicated on the degree of consistency with expectations [21]. Contextual integrity also suggests that these norms should not only consider the societal perspectives and values but also account for the individual interests and preferences of the affected parties. To understand these norms, numerous studies have explored patient willingness to consent to share PHI [9,13,22]. Factors related to willingness include individuals’ perspectives on the privacy and security of their PHI, the relevance of sharing, and how sharing PHI would directly affect the quality of care [9].

Patient willingness to share PHI to support advancements in health care is improved when there is transparency, especially when the information regarding the collection, use, and disclosure of their PHI is clearly stated [22]. Moreover, this transparency is often seen as a mechanism for improving the trustworthiness of digital health tools [22]. There are ongoing efforts to improve consent processes by providing patients with improved transparency on PHI management, enabling them to make more informed and meaningful choices about PHI sharing [16,23]. Innovative consent processes through the use of electronic consent (eConsent) have become common practice by health care organizations and digital health service providers for obtaining consent. eConsent refers to the use of electronic information systems (ie, multimedia resources such as infographics, videos, and embedded links) to convey information commonly found in a paper-based consent form such that an individual’s consent is obtained electronically [24,25]. With the growing use of eConsent, new consent models have emerged, each providing individuals with varying control and autonomy over the collection, use, and sharing of their PHI. Broad consent models [26] (ie, consenting to the use of PHI for broad purposes) have commonly been used by digital health service providers; however, newer consent models such as dynamic consent [26] (ie, providing consent through a web-based platform, with the ability to set or change consent preferences) are being adopted to better support meaningful and informed consent practices. The Office of the Privacy Commissioner (OPC) of Canada has deemed meaningful consent a vital component of Canadian privacy legislation, citing the importance of meaningful consent and choice in establishing and sustaining public trust in digital health [27,28]. This review explores patient perspectives and preferences regarding digital health consent. Patient experiences with digital health consent will also be explored to generate insights into optimizing the effectiveness of digital health consent processes, making consent meaningful for patients.

## Methods

### Purpose and Objectives

State-of-the-art reviews are intended to summarize emerging trends and synthesize insights from the most current literature [29]. This state-of-the-art review was conducted to understand the current state of research concerning patient perspectives on digital health consent. Understanding the current state is essential as research on consent is primarily driven in the context of understanding its role in participant recruitment [30], biobanks [31,32], registries [33,34], and secondary use [35,36], but there has been limited attention paid to consent within a broader digital health context [17]. The objectives of this review were to characterize the state of evidence, explore what is known about the patient perspective and experience with digital health consent, and provide recommendations on designing and implementing digital health consent based on the findings.

### Search Strategy

A structured literature search was developed and deployed in 4 electronic databases: MEDLINE, IEEE Xplore, Scopus, and Web of Science. The initial literature search was conducted in March 2021 and updated in March 2022. Primary peer-reviewed articles published after January 2010 were included in the search. The search strategy was first developed using Medical Subject Headings and keywords in MEDLINE and then translated to the other databases. Key search terms used in each database included *information systems*, *electronic health/medical records*, *telemedicine*, *telehealth*, *mobile apps*, *mHealth*, *digital/virtual health*, *artificial intelligence*, *consent*, *informed consent*, *eConsent*, *consent model*, *consent framework*, *consent pathway*, *consent requirement*, *consent standards*, *eGovernment*, and *eServices*. The search terms were combined using Boolean logic operators (eg, *digital health* OR *eConsent* AND *consent* OR *framework*). Although the search terms used were quite broad in nature, we narrowed our search to specifically include papers that described digital health interventions or innovations and consent preferences, behaviors, or experiences of patient populations. The full search strategy is presented in Multimedia Appendix 1.

### Selection Criteria

The search results were uploaded to Covidence, a literature screening and data extraction tool. In total, 2 reviewers (NS and IK) piloted the extraction tool with the first 50 articles and independently screened the titles and abstracts for relevancy afterward. Any discrepancies or conflicts were discussed and resolved. A total of 3 reviewers (IK, DI, and HB) independently assessed the full text of all relevant articles. Articles were flagged for discussion if it was unclear whether they met the inclusion criteria. In total, 2 authors (IK and JK) repeated these steps when updating the search.

Articles were included if they met all the following criteria: (1) described an eConsent or consent process, design, or development; (2) focused on patients’ perspectives, preferences, acceptance, or behaviors; (3) were set in a digital health or health IT context; and (4) described the use of digital health or health IT or digital PHI for health care delivery, health research or analytics, or consumer use. Study eligibility was not limited by study design, thereby allowing for the inclusion of various study designs. Only studies published in English were eligible for inclusion. Studies that did not meet the inclusion criteria were excluded from the review.

### Data Extraction and Analysis

A standardized data extraction form was developed on REDCap (Research Electronic Data Capture; Vanderbilt University), a secure software database for storing research data [37]. In total, 4 researchers (IK, DI, HB, and JK) independently extracted data from each article using a standardized data extraction template. Once extraction was complete, the full research team reviewed the compiled data. Three broad data collection categories were extracted from the articles: (1) study characteristics, (2) consent model, and (3) study results and main findings.

The extracted study characteristics included study title, year of publication, country of origin, sample size, sample source (eg, research, biobank or patient participant, and national or regional sample), study context or setting, study method, and study design and objectives. Data on consent models were extracted and categorized based on the seminal eConsent model framework by Coeira and Clarke [38]. The framework was then expanded to include contemporary models [26,39-42]. The types of consent models include *broad open*, *broad controlled*, *broad tiered/menu/meta*, *dynamic*, and *general denial consent*. The main findings of each study were extracted verbatim if they provided statistical analysis of patient perspectives and experiences with digital health consent.

A qualitative content analysis [43] of the free-text data was conducted using Microsoft Excel (Microsoft Corp). The analysis used an inductive approach in which the research team created codes and categories over several coding sessions. Once the coding was complete, the lead and senior authors (IK and NS) reviewed and refined the codes. Frequencies and percentages were calculated using Microsoft Excel.

A detailed overview of the included studies is presented in Multimedia Appendix 2 [44-116].

## Results

### Overview

**Figure 1:**
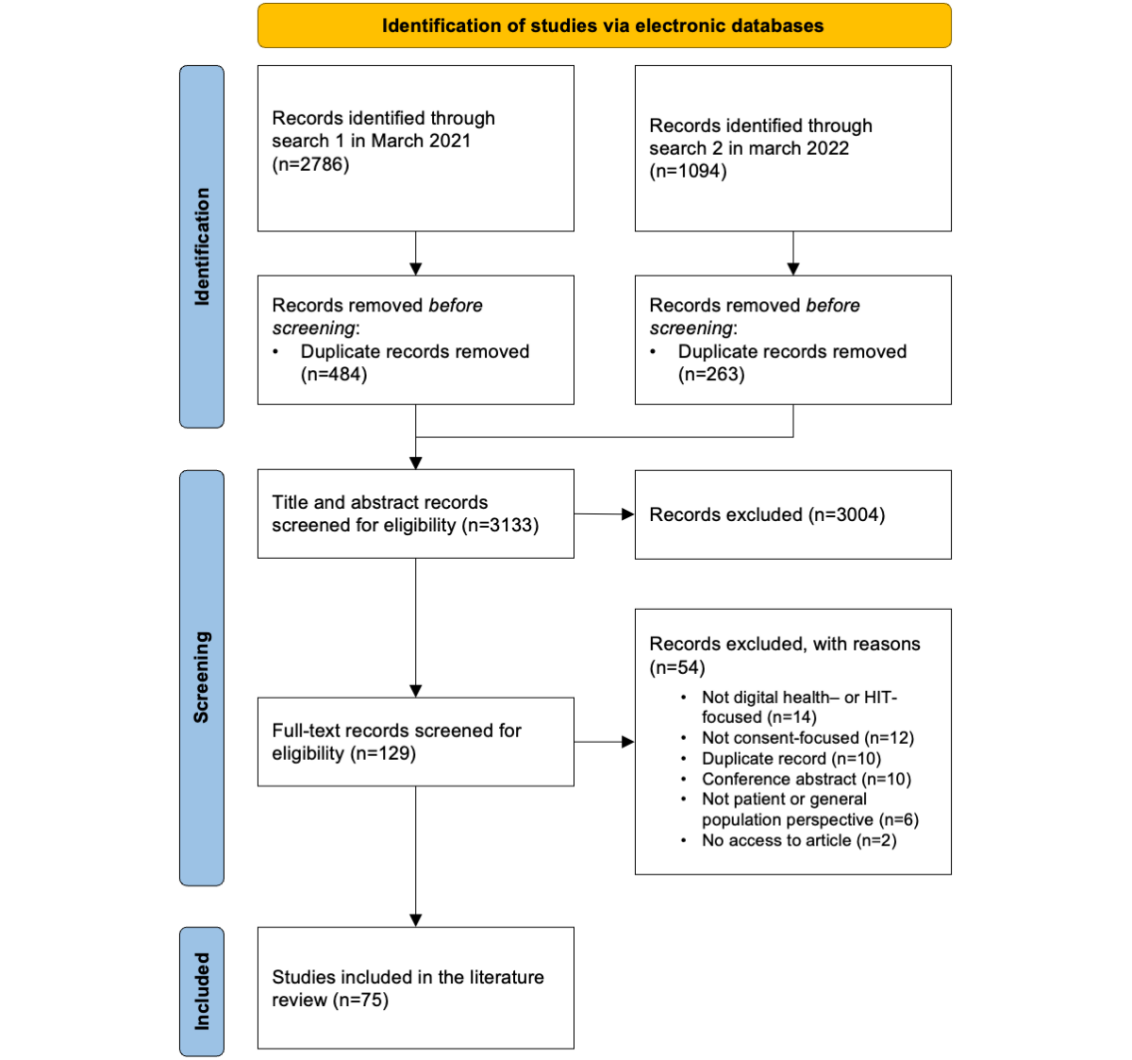
Article selection flowchart. HIT: health IT.

The search strategy yielded 3133 unique citations. Following the screening process, 2.39% (75/3133) of the articles were eligible for extraction. The flowchart of the search selection process can be found in Figure 1.

### Study Characteristics

Of the 75 studies included in this review, 58 (77%) were published within the last 5 years (between 2017 and 2022), and 48 (64%) were conducted in the United States. Most studies were conducted within a clinical care (33/75, 44%) or research context (29/75, 39%) where the study sample of focus was primarily research, biobank, and patient participants (54/75, 72%). Nearly half of the studies (36/75, 48%) used quantitative research methods, commonly using cross-sectional surveys (35/75, 47%) to collect data. The primary purpose of most studies was to understand participants’ willingness to share PHI (25/75, 33%) and participants’ perceived usability and comprehension of an eConsent platform (25/75, 33%). More than half of the studies (39/75, 52%) focused on participants’ hypothetical views on digital health consent. A total of 21% (16/75) of the studies described the design or development of an eConsent platform. In total, 27% (20/75) of the studies described the implementation, usability, or evaluation of an existing eConsent platform. Further details about the study characteristics are shown in Table 1.

**Table 1:** Study characteristics (N=75).

Characteristic		Studies, n (%)
**Year of study**		
	2010-2013	4 (5)
	2014-2016	13 (17)
	2017-2019	26 (35)
	2020-2022	32 (43)
**Country of origin**		
	United States	48 (64)
	England	6 (8)
	Ireland	3 (4)
	Sweden	3 (4)
	Germany	3 (4)
	Other^a^	12 (16)
**Study context**		
	Clinical care	33 (44)
	Research	29 (39)
	Consumer innovations	13 (17)
**Study purpose**		
	Willingness to share PHI^b^	25 (33)
	Usability and user comprehension	25 (33)
	Willingness to participate	12 (16)
	Consent information needs	6 (8)
	eConsent^c^ design and implementation	7 (9)
**Study methods**		
	Quantitative	36 (48)
	Mixed methods	23 (31)
	Qualitative	14 (19)
	Multimethods	2 (3)
**Study design**		
	Cross-sectional survey	35 (47)
	Focus groups	11 (15)
	Randomized controlled trial	7 (9)
	Interviews	4 (5)
	Multiple methods	12 (16)
	Other	6 (8)
**Study sample or population**		
	Research, biobank, or patient	54 (72)
	General population^d^	19 (25)
	Knowledge users	2 (3)
**Sample subgroup analysis**		
	No	57 (76)
	Yes^e^	18 (24)
**Presence of an eConsent platform**		
	Yes, they are developing one	16 (21)
	Yes, there is one that exists	20 (27)
	None used	39 (52)

^a^Country of origin: countries categorized as *Other* included Australia (2/75, 3%), Canada (2/75, 3%), South Korea (2/75, 3%), Switzerland (2/75, 3%), Colombia (1/75, 1%), Denmark (1/75, 1%), India (1/75, 1%), and Singapore (1/75, 1%).

^b^PHI: personal health information.

^c^eConsent: electronic consent.

^d^General population can be further divided into studies that focus on a national population (8/75, 11%) or on a regional-, provincial-, or state-level population (11/75, 15%).

^e^Sample subgroup analysis: *Yes*—studies conducted a subgroup analysis to understand whether participant demographic characteristics (eg, race and ethnicity, education, age, digital and health literacy, income, and sex and gender) affected their consent preferences and behaviors.

### Research Question 1: What Are Patient Preferences on Consent Models for Digital Health?

Table 2 presents a typology of consent models, outlining the models used across the studies. More than half of the included studies (40/75, 53%) did not describe or report the consent model used. Broad open consent was the most explored model (11/75, 15%) [44-54], followed by dynamic consent (8/75, 11%) [55-62], broad controlled consent (4/75, 5%) [63-66], broad tiered/meta/menu consent (3/75, 4%) [67-69], and general denial consent (2/75, 3%) [70,71].

**Table 2:** Spectrum of consent models reported (N=75).

Type of consent	Frequency, n (%)
Broad open—existing data and PHI^a^ do not require additional consent for use	11 (15)
Broad controlled—if consent is provided, data will only be used by approved investigators	4 (5)
Broad tiered, menu, or meta—consent process allows participants to select the types of research for which their PHI can be used	3 (4)
Dynamic consent—consent process allows participants to set and change their consent preferences through a secure platform	8 (11)
General denial—consent is required by participants on a per-use basis	2 (3)
Multiple consent models reported	7 (9)
Consent model not reported	40 (53)

^a^PHI: personal health information.

In total, 9% (7/75) of the studies compared various consent models to understand whether a specific model increased participants’ comprehension of what they were consenting to or made them more willing to consent. Within these studies, patient consent model preferences varied; however, in most studies (5/7, 71%), participants preferred granular, informative, and transparent consent choices [72-76]. For example, Kim et al [76] found that 76.6% (955/1246) of the participants made sharing choices to select at least one PHI value that they would not want to share with a particular researcher. Participants also noted that, if consent choices were not offered, they were less likely to share their PHI. Kaufman et al [72] found that, when presented with various consent models, participants had similar preferences for *general denial* (1873/2601, 72%), *broad tiered/menu/meta* (1951/2601, 75%), and *dynamic* consent (1899/2601, 73%). A *broad open* consent model was the least preferred among participants, with 64% (1665/2601) stating that they would be willing to share their PHI under this model.

The remaining 29% (2/7) of the studies found that participants preferred *broad open* consent. Riordan et al [77] found that broader consent models may be appropriate under specific circumstances, where 91% (2873/3157) of respondents expected to be explicitly asked for consent for their identifiable records to be accessed for health provision, research, or planning, whereas half (1547/3157, 49%) of the respondents expected to be asked for consent if their records were deidentified. Brown et al [78] found that more participants (19/31, 61%) preferred a *broad open* consent model over a *broad tiered* consent model (10/31, 32%) when agreeing to the secondary use of their biospecimens. Participants least preferred “notice consent” (ie, *general denial* consent), stating that it was not informative and did not invoke a sense of control over their PHI sharing preferences.

### Research Question 2: What Is Known About the Patient Perspective and Experience With Digital Health Consent?

In total, 91% (68/75) of the studies described the patient perspective or experience with digital health consent. These studies were further categorized according to their primary purpose (as described in Table 1): (1) user comprehension of consent notices and consent information needs (31/68, 46%), (2) willingness to consent to participate (12/68, 18%), and (3) willingness to consent to share PHI (25/68, 37%).

#### User Comprehension of Consent Notices and Consent Information Needs

In nearly half (15/31, 48%) of the studies, comprehension was assessed by comparing how different consent media such as eConsent or paper-based consent affected participants’ understanding of the consent information. A total of 73% (11/15) of the comprehension studies found that user comprehension improved when an eConsent medium was used. Participants in a randomized controlled study reported a greater understanding of most aspects of the consent notice in an eConsent platform than in a paper-based consent form [79]. Another study found that participants who used video- or app-based eConsent had greater comprehension than those provided with paper-based consent [80]. A longitudinal study [53] comparing understanding of 3 eConsent mediums with varying degrees of customizable information (eg, options to visit hyperlinks for additional information) and messaging found that participant understanding was similar among the 3 versions at the 1-week follow-up; however, participants with the least customizable eConsent version had a significant decrease in understanding at the 6-month follow-up.

Improvements in user comprehension of consent notices were attributed to the overall user satisfaction with and usability of eConsent systems. These systems were described as easy to use, well organized, and more engaging [47,50,53,54,58,80-89]. Specifically, a video eConsent system was favored in 7% (1/15) of the studies as participants felt that they could move forward through the video at their own pace, improving their understanding of what they were consenting to [82]. Other studies (21/31, 68%) exploring the use of different eConsent media such as web-based portals [48,53,89-91], animated videos and visuals [54,79,80,88,92,93], tablet kiosks [47,81], graphic organizers and mind maps [94], and mobile apps [56,58,62,69,85,95,96] found that participants favored the customizable elements (eg, drop-down menus, buttons, links, and multimedia) of these eConsent formats as they were more engaging and improved the presentation of consent information.

Among 19% (6/31) of the studies, mixed results were identified when assessing the amount of information and information elements that individuals require when reviewing consent notices. Beskow et al [44] found that information needs to consent to a hypothetical biobank differed at the individual level. Although 61% (34/56) felt that the information presented in the 2-page simplified and concise consent form supported their decision-making, 39% (22/56) of the participants wanted more information in the consent form. Another study found that providing additional information within their consent form did not discourage participation in a digital trace data collection app as there were no significant differences in rates of consent between participants who clicked to view a description of an app function and those who did not [65]. A few studies (3/31, 9.7%) suggested providing participants considered to be “high information seekers” with the option or ability to drill down on information elements within a consent form (ie, supplementary information and click to expand) [44,49,65]. Information elements deemed most important to participants included the purpose of the proposed initiative, duration of the study, permitted uses and access, data handling and control, safeguards and security measures, and the risks and benefits of consenting [49,60,65,97,98].

#### Willingness to Consent to Participate

In total, 18% (12/68) of the studies described participants’ willingness to consent to take part in an initiative or intervention (eg, research, clinical care, consumer digital health innovation, and biobank). Of the 12 studies, 5 (42%) found that most participants were willing to consent to participate. However, willingness to consent was contingent on several factors. Most often, willingness to consent depended on who their PHI would be shared with, where many participants were less trusting of entities outside their circle of care. For instance, the more trusting the participants were in their health care provider, the less control they required over their PHI [51,74,75]. Moreover, Deverka et al [74] gathered user requirements from stakeholders to support the design and management of a Medical Information Commons. The stakeholders expressed that granting for-profit entities access to participants’ information would reduce participation and trust in the Medical Information Commons. Sanderson et al [73] found that, although many participants trusted the health care system (8141/13,000, 63%) and medical researchers (7748/13,000, 60%), those reporting lower trust in the health care system and medical researchers were less willing to participate in the biobank. In interviews with users of a mobile health app, Zhou et al [99] found that, when participants were asked whom they would like to share their PHI with, 93.2% (109/117) indicated that they would share their PHI with their health care provider, 69.2% (81/109) would share it with family members, and 32.5% (38/117) would share it with friends. Another study identified a greater reluctance among participants to share PHI with pharmaceutical company researchers (1353/2601, 52%) and government researchers (1144/2601, 44%) [72]. Overall, the importance of building and fostering participant trust in digital health initiatives was described, calling for greater transparency in consent practices [75,100].

Participant concerns related to the privacy of their PHI and the perceived sensitivity of their PHI also hindered their willingness to participate. In a focus group study [51], participants raised concerns about the privacy and confidentiality of their biospecimens and EHR data. These concerns were primarily related to how their PHI was stored, protected, and deidentified. In a survey conducted by Sanderson et al [73], 90% (11,397/13,000) of participants agreed that health information privacy was important to them, and 64% (8135/13,000) agreed that they were worried about the privacy of their health information. Furthermore, individual privacy concerns were associated with a greater need for control over biospecimens among women participating in a breast cancer biobank study [75]. Finally, Cavazos-Rehg et al [101] explored how parental consent requirements would affect adolescents’ willingness to participate in a mental health app. Although 35% (106/303) of adolescents indicated that they would be willing to allow researchers to contact their parents for consent, 30% (91/303) would not allow researchers to contact their parents. This was primarily attributed to the importance of retaining privacy and autonomy.

Participation in the aforementioned initiatives was dependent on a variety of sociodemographic factors. A total of 42% (5/12) of the studies examined the effect of sociodemographic characteristics on willingness to consent to participate, finding that age [72,99], income [99], education [72,73], race [73,75,78], and religious beliefs [73] affected consent decisions. Participants who identified as racialized persons were less likely to consent when compared with participants identifying as White [73,75]. Consent decisions often depended on the granularity of consent such that those identifying as racialized persons preferred granular consent options (ie, control over PHI collection, use, and sharing) instead of broad consent options [78]. Furthermore, Sanderson et al [73] found that those who had less education and were religious were less willing to consent. In comparison, those highly educated [72] and younger [72,99] were more willing to consent. Zhou et al [99] also found that participants who made <US $10,000 annually exhibited the least concerns about the security and privacy of their PHI. In contrast, participants who made >US $75,000 annually expressed the strongest concerns and desire for security and privacy of their PHI.

#### Willingness to Consent to Share PHI

A total of 37% (25/68) of the studies explored participants’ willingness to consent to sharing their PHI for clinical care, research, biobanks, precision medicine initiatives, and consumer innovations. Generally, study participants were willing to share their PHI under certain conditions. For example, participants expressed greater comfort and willingness to share their PHI with health care providers, academic researchers, and not-for-profit organizations [46,55,57,64,71,102-109]. Participants were reluctant to share their PHI with for-profit organizations, pharmaceutical companies, government organizations, and researchers [46, 55, 57, 64, 71, 103, 105-108, 110]. A focus group study exploring prospective genome research and repositories found that participants generally endorsed the value of sharing their PHI, especially with academic health researchers and nonprofit organizations; however, participants expressed apprehension toward sharing with for-profit entities because of the belief that for-profit entities would use their PHI to generate financial returns [105]. This finding was echoed in an American survey study in which individuals who tracked their PHI were willing to share it for health research and were more trusting of academic researchers than of for-profit entities [106].

A lack of information and transparency surrounding PHI-handling practices hindered participants’ willingness to provide consent. Unwillingness was most often attributed to a lack of information on the anonymization, aggregation, or deidentification of PHI [71,77,105,107,111,112]; the privacy policies and auditing practices of entities [105,106,112,113]; and consent or data-sharing options [55,57,71,76,108,114]. Participants’ willingness to share their PHI depended not only on whom they were sharing it with but also on how their PHI was to be used and for what purpose. Belfrage et al [109] found that most survey participants would not allow their EHR data to be used for quality assurance, research, or clinical education. In contrast, those who were more trusting of the health care system were more willing to permit these uses. Another study found that 79% (100/126) of participants would be willing to share their EHR data for research purposes, and 73% (92/126) indicated that knowing who would be accessing their EHR data would make them more comfortable in sharing them [57]. Grande et al [110] found that the specific use of an individual’s PHI influenced their willingness to share it for secondary purposes more than the user of the PHI and the sensitivity of the PHI. A focus group study also found that participants were more likely to share their mobile phone location data with health agencies if provided with information on how their data would be used, stored, and protected [111]. Overall, providing participants with further information about who can access their PHI and how entities can use it invoked a greater sense of trust in sharing PHI [102,103,111,113].

Several antecedents that either supported or hindered consent decisions were identified in the studies. Specifically, past health care and privacy experiences and health care perceptions influenced willingness to consent. Weidman et al [55] found that participants who had previously undergone a genetic test were more willing to share their PHI than those who had not. Moreover, participants with high levels of distrust in the health care system and those without a usual source of care were less supportive of secondary uses of their electronic PHI [110]. A Swedish health system user survey also found that participants with a self-reported health status of “good or very good” had higher trust in the health care system than those with a “bad or very bad” self-reported health status [109]. A focus group study by Murphy et al [111] found that, after headlines about the Cambridge Analytica scandal emerged, participants acknowledged a greater responsibility toward protecting their PHI; however, many participants did not act to safeguard their PHI further. A recurring theme was the inevitability of PHI being accidentally released or breached, raising concerns about whether privacy and security can be guaranteed by the entities who collect their PHI [105].

Across the studies, health care perceptions and consent decisions were often driven by altruistic beliefs [55, 67, 71, 103, 105, 107, 115]. For instance, participants in an observational study wearing mobile imaging and pervasive sensing and tracking devices did not consider privacy a primary concern [67]. Although 35% (29/82) reported having extremely private preferences or expectations for privacy, their participation in the study was motivated by the positive contributions this research could have toward health sciences, outweighing a temporary loss of privacy [67]. Rivas Velarde et al [103] found that, among focus group participants, their decisions to share their PHI for research were driven by the contributions the research could make toward the greater public good. Altruism was further demonstrated by Spencer et al [55] and Rowan et al [115], finding that most study participants understood the importance of sharing their PHI for research purposes, for the benefit of medical progress, and for the benefit of society.

It was also found that expectations regarding consent varied with sociodemographic factors and digital literacy. A UK study found that racialized participants with less education and lower digital literacy were more likely to prefer to be asked for explicit consent before their deidentified health records were accessed [77]. Consent preferences also differed by age group, where younger participants were less likely to consider informed consent important [116]. A survey of veterans enrolled in a US-based health care organization found that respondents who identified as White, male, and less educated were more likely to endorse information sharing without the need for consent [52]. Participants aged >60 years and those deemed to have an adequate health literacy level were more willing to share more items in their EHR than younger participants or those who did not report having an adequate health literacy level [76].

## Discussion

### Principal Findings

Consent in digital health is contextually driven such that it is often dependent on who is using or accessing one’s PHI, how their PHI will be used, and for what purpose. The findings of this review underscore the context dependency of consent as there were mixed results on patient perspectives on consent models and willingness to share their PHI. For instance, broad consent models may be acceptable in specific study contexts. In contrast, consent models that provided patients with more control were favored in others (ie, broad tiered, menu, or meta and dynamic consent). Similarly, patient willingness to share their PHI, consent behaviors, perceptions, and preferences varied by study. Given this variance, enabling individuals to make informed choices based on their contexts is critical. At the most rudimentary level, individuals require specific and easily comprehensible information on who their PHI is being shared with, for what purpose their PHI will be used, and how the privacy and security of their PHI will be ensured [10,117]. Providing individuals with adequate information to make an informed choice fosters transparency—the moral and ethical obligation to enable meaningful consent [72,97,117,118]. The insights gathered and summarized in this review highlight the need to recognize individual behaviors and preferences when designing and implementing the consent processes of digital health initiatives and the importance of building and sustaining trust and transparency.

### Consent Behaviors, Preferences, and Perceptions

Trust is central to individual consent behaviors, preferences, and perceptions. Willingness to consent often depended on the entity collecting PHI, where most individuals were comfortable sharing PHI with their health care providers, health care organizations, and academic researchers. Comfort in sharing PHI declined with recipients outside the individual’s circle of care, particularly with commercial or for-profit entities. There is a growing body of evidence highlighting individuals’ significant discomfort in sharing their PHI with commercial and for-profit entities, primarily because of a lack of trust in these entities [10,13,119-121]. This discomfort has been predominantly attributed to privacy concerns, loss of control and autonomy over one’s PHI, and the potential for misuse of one’s PHI (ie, for monetary gains) [13,19,121,122]. There was an evident desire for individuals to have greater control over their PHI-sharing preferences, largely attributing these needs to past privacy experiences, health care experiences, and general health care perceptions. Specifically, this review found that those with positive experiences within the health care system and those with access to a trusted usual source of care were more willing to share their PHI [110,112].

In contrast, those with poor health care experiences and awareness of commercial entities misusing PHI were less willing to share their PHI [105,111,112]. Unsurprisingly, other studies have found mixed results on how past health care and privacy experiences affect intentions to share [10,19,123]. This supports the notion that privacy concerns are contextually driven such that individual experiences, environmental factors, and personal dispositions influence consent behaviors and attitudes [124]. Thus, to build trust in sharing PHI outside the circle of care, understanding the influence of these past experiences on individual privacy concerns warrants further consideration and research [19,123].

Although many individuals were concerned about the potential risks of consenting and the confidentiality of their PHI, in several studies, the benefits outweighed the potential risks [55,67,71,103,105,107,115]. There was a general willingness to share PHI in support of research efforts and improvements to health care outcomes so long as the benefits of sharing were clear [19]. A sense of social responsibility and altruistic beliefs about improving care and treatment outcomes for oneself and society prevailed. A common explanation for this behavior is the privacy calculus, where an individual’s information-sharing decisions are based on a weighing of future consequences related to the benefits and risks of sharing [125]. For instance, Kaufman et al [126] found that general concerns about protecting one’s privacy were not substantially related to their willingness to participate in a biobank. This discordance or privacy paradox is echoed in a systematic review of patient privacy perspectives on health information exchange [124], concluding that studies are increasingly finding that individuals often rationalize the risks of sharing information by considering the potential benefits (ie, privacy calculus).

### Consent Management and Information Needs

The need to modernize consent processes for the digital age is widely recognized as legislation has not kept pace with the rapidly evolving digital health environment [10,27,117]. The implementation of consent processes that adequately reflect and incorporate end users’ needs has been slow and insufficient. To modernize consent processes, *dynamic* consent models have been commonly adopted to allow individuals to update or alter their consent preferences when needed [127]. *Dynamic* consent has been described as a means to improve individual autonomy by enhancing choice, comprehension, and engagement in the consent process [26,41]. Although dynamic consent may enhance informed choice, studies in this review more commonly explored or implemented *broad* consent models. The rationale for implementing *broad* consent models has been attributed to the ease of implementation, the minimal impediment to the progression of research, and the lowered risk of “consent fatigue” [26,39]. From the patient perspective, this study found that consent models that offered enhanced control and options over their PHI were preferred over a *broad* consent model [35,72-76].

Interestingly, this review also found that the type of consent model may have little relevance to participants’ decisions to consent [73,75]. Instead, consent decisions depended on whether individuals felt well informed and trusted with whom they shared their PHI. For instance, Soni et al [108] found that, although mental health information was deemed sensitive among participants, they were still willing to share it depending on who the provider was (eg, behavioral vs nonbehavioral health care provider). This adds to the notion that preference for data sharing is not solely tied to the type of data being shared but instead is intrinsically associated with past experiences (ie, stigma and discrimination) and trust in the data recipient [108]. This finding is especially salient as the mental health privacy discourse is heightened by special legislation and expectations grounded in the subjective sensitivity of the PHI—often contributing to the disconnect between the historical paternalistic approach to protecting patient privacy and their nuanced data-sharing preferences [128]. Improving transparency around data sharing and the impacts of sharing may empower these individuals to better contextualize their past experiences, thereby supporting greater autonomy in data-sharing decisions and trust in the data recipients.

In terms of transparency, there was mixed evidence on the best practices for presenting information on consent forms. The mixed evidence is characterized by a dichotomy, where some assert that more detailed information better supports consent decisions [129], whereas others contend that brevity supports meaningful consent [130-132]. However, this review also found suggestive evidence that the quantity of information presented in a consent notice is less important when considering the quality of the information presented (ie, clear, transparent, and informative) [75,100,102,103,111,113]. As society becomes increasingly digital, consent design considerations should stem beyond document length and instead prioritize innovations in the presentation of consent information [117]. As illustrated in this review, customizable electronic formats can facilitate more informed consent decisions (eg, links, drop-down menus, and multimedia) [30,36]. Consistent with the meaningful consent guidelines of the OPC, this customizable approach would allow individuals to control how much information they wish to process, thereby tailoring the consent form to support an informed decision [28]. Future research should focus on understanding individual consent requirements when designing and developing eConsent platforms, aiding in implementing more meaningful functionalities.

### Contributions, Future Research, and Limitations

This review provides insights into patient consent preferences in the digital health context. Given the rapid adoption and integration of digital health technologies in clinical care settings, it is unsurprising that many of the included studies were published within the last 5 years (2017 to 2022; 58/75, 77%). The summative findings of this review present the current state of patient consent preferences and emerging consent practices in the digital health context. Currently, most studies focus on collecting and using electronic PHI and EHR data for biobanks and research initiatives. Few studies (6/75, 8%) focused on understanding patient preferences, behaviors, and perspectives on consent in AI (ie, precision medicine, machine learning, and deep learning). As AI becomes more pervasive in clinical predictions and diagnosis, treatment recommendations and decision support, and consumer health innovations, additional research is needed to explore individuals’ consent preferences and experiences in these contexts.

Consistent with state-of-the-art reviews, this study highlighted gaps in digital health consent research. Although the included studies explored patient or public consent preferences, many (40/75, 53%) failed to clearly outline the type of consent model used. Reporting the consent model is essential as it provides greater context to the study findings, especially concerning individual perceptions. As with privacy research [124,133], more than half (39/75, 52%) of the studies relied on hypothetical scenarios to understand participants’ consent decisions. Although these studies generate insights that can inform how to approach consent for digital health initiatives, they only represent intentions rather than behaviors. They are subject to the privacy paradox [10,124]. Moreover, this review found that there were fewer qualitative and mixed methods studies. These formative or needs assessment studies may benefit from qualitative and mixed methods approaches to better understand individual preferences and behaviors [124].

When considering how health equity has become increasingly important in health care research [134], it was surprising to see the lack of publications exploring consent with a health equity lens. Such studies analyzed how various sociodemographic factors affected participants’ consent behaviors and preferences, finding that race, age, income, and education individually contributed to consent and data-sharing preferences. However, these findings do not provide enough depth to understand the collective, intersectional factors that influence these decisions [10]. By viewing these factors individually, the underlying and multifaceted impacts of trust and antecedents on consent behaviors and decisions are not adequately acknowledged [133]. Future research must extend beyond the notion that individual characteristics such as race and age are in themselves associated with consent decisions and examine the influences of past and current experiences (eg, distrust in the health care system and negative health care and privacy experiences) on consent behaviors [71,135,136]. In doing so, we may uncover valuable insights into building trust and empowerment within these groups.

Finally, the findings of this review highlight important considerations for designing consent in the digital health era. The design of meaningful consent processes must be rooted in co-design approaches, transparent practices, and integrated knowledge translation. As echoed by the OPC Meaningful Consent Guidelines [28], stakeholder and consumer perspectives must be included in the design of consent, where co-design with the general public, policy decision makers, digital health service providers, and graphic designers is needed. This will ensure that diverse needs and requirements are met throughout the design, development, and implementation of consent processes. Moreover, the transparency of consent notices was a reoccurring theme in this literature review, where willingness to consent was contingent on how informed individuals felt. Transparency can be facilitated by ensuring that information presented within consent notices is customizable, comprehensible, and accessible [117]. Furthermore, in progressing the call for meaningful consent in digital health, there is a strong need to ensure that knowledge dissemination and translation efforts are prioritized, especially with regard to sharing best practices and lessons learned from the use of eConsent platforms and consent models for digital health innovations.

There are some limitations to consider, most of which are related to the state-of-the-art review methodology [29,137]. State-of-the-art reviews intend to provide a snapshot of the most current literature on a given subject. Given the time-bound nature of this review methodology, it is possible that we did not include all the available literature. To mitigate this, we deployed our search strategy in several multidisciplinary academic search engines to obtain a comprehensive review of the literature. Furthermore, a quality assessment of the included articles was not conducted as it was beyond the scope of this review methodology. The search strategy and screening methodology should also be considered when interpreting the results. For instance, in this literature review, we searched only in MEDLINE-indexed journals as opposed to searching in PubMed-indexed journals, resulting in potentially relevant articles not being included in our search. Furthermore, by limiting the search of the literature to primary research studies published in the past decade, we may have missed studies that may have been relevant for inclusion in this review. However, fewer than one-quarter (17/75, 23%) of the studies included in this review were published before 2016. Considering the rapid advancements in digital health within the past decade, studies published before 2010 may not have provided much insight. Finally, 64% (48/75) of the studies included in this review were conducted in the United States, which may limit the generalizability of our findings given the differing cultural, social, legislative, and political environments in other countries. In addition, given the finding that consent preferences among individuals are context dependent, there is a need for further research focusing on patient and public digital health consent preferences in other regional and national contexts.

### Conclusions

Consent is an increasingly important issue in the rapidly evolving digital health ecosystem. Implementing meaningful consent may be a complex endeavor as consent preferences and behaviors will vary based on context; however, this review found that most patients are willing to consent to share their PHI given the right circumstances. Suppose that the desired outcome is to use one’s PHI to develop, sustain, and enhance digital health innovations. In such cases, individuals must be provided with transparent information about the purpose of the collection and use of their PHI and the potential benefits, whether direct or indirect, of consenting to share their PHI. In addition to transparency, information must be customizable, allowing readers to tailor the granularity of detail to their individual needs. By enabling meaningful and informed consent, organizations can foster greater trust in their digital health solutions. Furthermore, to understand how to facilitate meaningful and informed consent in various contexts, patients and the public must be engaged in the design, development, and implementation of consent processes and notices for digital health initiatives. By doing so, consent practices in the digital health context will not simply act as a proxy for choice but will also be able to fulfill the notion of contextual integrity such that they account for individual interests and preferences in specific social contexts.

## Appendix

Multimedia Appendix 1 Literature search strategy.

Multimedia Appendix 2 Overview of the included studies.

## Abbreviations

AI: artificial intelligence
eConsent: electronic consent
EHR: electronic health record
OPC: Office of the Privacy Commissioner
PHI: personal health information
REDCap: Research Electronic Data Capture
