# Supplementary material for: Patient Perspectives and Preferences for Consent in the Digital Health Context: State-of-the-art Literature Review
Source: J Med Internet Res. 2023 Feb 10;25:e42507. doi: 10.2196/42507 (PMC9960046; doi:10.2196/42507)
Supplement: Multimedia Appendix 1 [file jmir_v25i1e42507_app1.docx]

Multimedia Appendix 1: Literature Search Strategy

Ovid MEDLINE: Epub Ahead of Print, In-Process & Other Non-Indexed Citations, Ovid MEDLINE® Daily and Ovid MEDLINE® <1946-Present>

1 exp Information Systems/ or electronic health records.mp. or exp Medical Records Systems, Computerized/ or exp Electronic Health Records/ or exp Hospital Information Systems/ or exp Medical Laboratory Science/ or exp Humans/ 20589720

2 telemedicine.mp. or exp Telemedicine/ 48353

3 precision medicine.mp. or exp Precision Medicine/ 37696

4 mobile applications.mp. or exp Computers, Handheld/ or exp Mobile Applications/ or exp User-Computer Interface/ 58361

5 (health* or Medicine).mp. [mp=title, abstract, original title, name of substance word, subject heading word, floating sub-heading word, keyword heading word, organism supplementary concept word, protocol supplementary concept word, rare disease supplementary concept word, unique identifier, synonyms] 4963969

6 (digital or electronic* or virtual).mp. [mp=title, abstract, original title, name of substance word, subject heading word, floating sub-heading word, keyword heading word, organism supplementary concept word, protocol supplementary concept word, rare disease supplementary concept word, unique identifier, synonyms] 612145

7 electronic health records.mp. or exp Medical Records Systems, Computerized/ or exp Electronic Health Records/ or exp Medical Laboratory Science/ or exp Hospital Information Systems/ or exp Information Systems/ 321740

8 "precision medicine".mp. or exp Precision Medicine/ 37696

9 "telemedicine".mp. or exp Telemedicine/ 48353

10 "mobile applications".mp. or exp Computers, Handheld/ or exp Mobile Applications/ or exp User-Computer Interface/ 58361

11 (health* or Medicine).mp. [mp=title, abstract, original title, name of substance word, subject heading word, floating sub-heading word, keyword heading word, organism supplementary concept word, protocol supplementary concept word, rare disease supplementary concept word, unique identifier, synonyms] 4963969

12 (digital or electronic* or virtual).mp. [mp=title, abstract, original title, name of substance word, subject heading word, floating sub-heading word, keyword heading word, organism supplementary concept word, protocol supplementary concept word, rare disease supplementary concept word, unique identifier, synonyms] 612145

13 11 and 12 162118

14 7 or 8 or 9 or 10 or 13 549603

15 artificial intelligence.mp. or exp Artificial Intelligence/ 156995

16 14 or 15 669966

17 Consent.mp. or exp Informed Consent By Minors/ or exp Informed Consent/ or exp Presumed Consent/ or exp Parental Consent/ 96666

18 limit 17 to (abstracts and structured abstracts) 39305

19 ("e-consent" or "electronic consent" or "eConsent").mp. [mp=title, abstract, original title, name of substance word, subject heading word, floating sub-heading word, keyword heading word, organism supplementary concept word, protocol supplementary concept word, rare disease supplementary concept word, unique identifier, synonyms] 143

20 "consent model".ab,kf,ot,ti. 136

21 "consent framework".ab,kf,ot,ti. 15

22 "consent requirement*".ab,kf,ot,ti. 398

23 "consent pathway*".ab,kf,ot,ti. 19

24 "consent scenario*".ab,kf,ot,ti. 6

25 "consent indicator*".ab,kf,ot,ti. 0

26 "consent standard*".ab,kf,ot,ti. 80

27 "consent approach*".ab,kf,ot,ti. 99

28 "consent method*".ab,kf,ot,ti. 65

29 20 or 21 or 22 or 23 or 24 or 25 or 26 or 27 or 28 800

30 16 and 18 2281

31 16 and 29 72

32 19 or 30 or 31 2385

33 limit 32 to (English language and yr="2021 -Current") 508

Web of Science

TS=( ( ( ( digital OR electronic* OR virtual ) near/3 ( medicine OR health* ) ) OR e-health* OR emedicine OR tele-health* OR tele-medicine OR telehealth* OR telemedicine OR ( ( precision OR personalized OR individualized ) near/3 AND medicine ) OR ( electronic AND near/3 ( health OR medical OR patient ) near/3 AND record* ) OR ( mobile AND near/3 AND health* ) OR m-health* OR mhealth* OR ( ( health OR medical ) near/3 ( app OR apps OR application* ) ) OR ( ( ( mobile OR cell** AND near/3 ( device* OR phone* ) ) OR smartphone* OR iphone OR ipad OR tablet OR wearable* ) AND ( medic* OR health* ) ) ) ) OR TS= ( ( e-service* OR eservice* OR "electronic service" ) OR ( e-government OR egovernment OR "electronic government" ) OR ( econsent OR e-consent OR "electronic Consent" ) ) AND TS= ( consent OR ( consent AND near/3 ( framework* OR model* OR pathway* OR requirement* OR standard* ) ) )

Scopus

TITLE-ABS-KEY ( ( ( ( digital OR electronic* OR virtual ) near/3 ( medicine OR health* ) ) OR e-health* OR emedicine OR tele-health* OR tele-medicine OR telehealth* OR telemedicine OR ( ( precision OR personalized OR individualized ) near/3 AND medicine ) OR ( electronic AND near/3 ( health OR medical OR patient ) near/3 AND record* ) OR ( mobile AND near/3 AND health* ) OR m-health* OR mhealth* OR ( ( health OR medical ) near/3 ( app OR apps OR application* ) ) OR ( ( ( mobile OR cell** AND near/3 ( device* OR phone* ) ) OR smartphone* OR iphone OR ipad OR tablet OR wearable* ) AND ( medic* OR health* ) ) ) ) OR TITLE-ABS-KEY ( ( e-service* OR eservice* OR "electronic service" ) OR ( e-government OR egovernment OR "electronic government" ) OR ( econsent OR e-consent OR "electronic Consent" ) ) AND TITLE-ABS-KEY ( consent OR ( consent AND near/3 ( framework* OR model* OR pathway* OR requirement* OR standard* ) ) )

IEEE XPLORE

(((( consent NEAR/3 ( framework* OR model* OR pathway* OR requirement* OR standard* ) ) )) ) OR ( econsent OR e-consent OR (electronic NEAR/3 Consent" ) )
